# Diagnostic Laparoscopy as a Selection Tool for Patients with Colorectal Peritoneal Metastases to Prevent a Non-therapeutic Laparotomy During Cytoreductive Surgery

**DOI:** 10.1245/s10434-019-07957-w

**Published:** 2019-10-22

**Authors:** Judith E. K. R. Hentzen, Reickly D. N. Constansia, Lukas B. Been, Frederik J. H. Hoogwater, Robert J. van Ginkel, Gooitzen M. van Dam, Patrick H. J. Hemmer, Schelto Kruijff

**Affiliations:** 1 Division of Surgical Oncology, Department of Surgery, University Medical Center Groningen, University of Groningen, Groningen, The Netherlands; 2 Division of Hepatopancreatobiliary Surgery, Department of Surgery, University Medical Center Groningen, University of Groningen, Groningen, The Netherlands; 3 Department of Nuclear Medicine, Molecular Imaging and Intensive Care, University Medical Center Groningen, University of Groningen, Groningen, The Netherlands

## Abstract

**Objective:**

The aim of this study was to evaluate the introduction of diagnostic laparoscopy (DLS) in patients with colorectal peritoneal metastases (PM) to prevent non-therapeutic laparotomies during cytoreductive surgery with hyperthermic intraperitoneal chemotherapy (CRS + HIPEC).

**Methods:**

Patients with histologically proven colorectal PM who underwent a laparotomy for potential CRS + HIPEC from January 2006 to January 2019 were retrospectively identified from a prospectively maintained database. In 2012, DLS was introduced in the preoperative work-up for CRS + HIPEC in our academic center. The rates of non-therapeutic laparotomies, major postoperative complications (Clavien–Dindo grade III or higher), and survival outcomes were investigated for patients who underwent a laparotomy before (cohort A) and after (cohort B) the introduction of DLS. In cohort B, the reasons to refrain from DLS were retrospectively explored from medical records.

**Results:**

Overall, 172 patients were included [cohort A: 48 patients (27.9%); cohort B: 124 patients (72.1%)]. A significant drop in the rate of non-therapeutic laparotomies occurred in cohort B compared with cohort A (21.0 vs. 35.4%: *p* = 0.044), despite only 85 patients (68.5%) from cohort B undergoing DLS in our academic center. The most important reason to refrain from DLS was a recently performed DLS or laparotomy in the referring hospital (48.7%). Major postoperative complications, in-hospital mortality, and survival outcomes were similar for both cohorts.

**Conclusions:**

Performing DLS during the preoperative work-up for CRS + HIPEC prevents non-therapeutic laparotomies in patients with colorectal PM. We recommend performing this laparoscopic screening in an experienced HIPEC center.

Worldwide, carefully selected patients with limited and resectable colorectal peritoneal metastases (PM) are treated with cytoreductive surgery (CRS) followed by hyperthermic intraperitoneal chemotherapy (HIPEC) with the aim of achieving long-term survival.^1^^–^5 Patients with low tumor burden, as expressed by the peritoneal cancer index (PCI), and in whom a complete cytoreduction of all macroscopic visible colorectal PM can be achieved (CC-0), benefit the most from this extensive surgical procedure in terms of survival.^5^^–^9 Therefore, CRS + HIPEC for patients with colorectal PM is restricted to those with a PCI ≤ 20, in whom a complete macroscopic cytoreduction can be reached.^8^^–^11

To date, surgical oncologists are still discovering the real extent and potential resectability of colorectal PM at the time of operative exploration, as current imaging modalities underestimate both important prognostic factors.^12^^–^14 Unfortunately, 20–40% of these patients are excluded for CRS + HIPEC directly after exploratory laparotomy, resulting in an open–close procedure (e.g. non-therapeutic laparotomy).^15^^,^^16^ For patients, this is a very undesirable postoperative outcome as it is not only associated with a significant risk of postoperative complications and a diminished quality of life (QoL) in the short term, but it also delays enrollment into other therapies. From a healthcare perspective, an aborted CRS + HIPEC procedure is expensive and leads to a longer wait list.

Suggestions have been made to use diagnostic laparoscopy (DLS) in the preoperative work-up for CRS + HIPEC to prevent non-therapeutic laparotomies during CRS in patients with colorectal PM.^17^^,^^18^ Several studies show that DLS is an accurate and safe staging tool in patients with peritoneal disease.^16^^,^^18^^–^23 However, the limitations of these studies are the variety of primary tumors that are included and the highly selected way a DLS is used. Since 2012, HIPEC surgeons from our academic center have introduced DLS as part of the preoperative work-up for CRS + HIPEC to prevent unnecessary laparotomies. This provides the opportunity to compare a historical cohort of patients with colorectal PM who were scheduled for CRS + HIPEC before the introduction of DLS with those with colorectal PM who were scheduled for CRS + HIPEC after DLS was part of the preoperative work-up. Our aim was to evaluate the implementation of DLS in the preoperative work-up for CRS + HIPEC, and the impact on preventing non-therapeutic laparotomies in this vulnerable population.

## Methods

### Design, Setting, and Patients

All consecutive patients with histologically proven colorectal PM who underwent an exploratory laparotomy for potential CRS + HIPEC from January 2006 to January 2019 were retrospectively identified from a prospectively maintained institutional database. Patients were divided into two different cohorts according to their operation date to evaluate the implementation and impact of performing DLS during the preoperative work-up for CRS + HIPEC to prevent non-therapeutic laparotomies. Study cohort A consisted of a historical group of patients with colorectal PM who underwent an exploratory laparotomy for potential CRS + HIPEC before the introduction of DLS in the preoperative work-up for CRS + HIPEC (January 2006 to December 2011), while study cohort B consisted of patients with colorectal PM who underwent an exploratory laparotomy for potential CRS + HIPEC after the introduction of DLS in the preoperative work-up for CRS + HIPEC (January 2012 to January 2019). The Ethics Committee of the University Medical Center Groningen approved this study (METc 201800395).

### Primary and Secondary Outcomes

The primary outcome was the rate of non-therapeutic laparotomies during CRS for cohorts A and B. Secondary outcomes were major postoperative complications, in-hospital mortality, disease-free survival (DFS), and overall survival (OS). Furthermore, to evaluate the implementation of DLS in the preoperative work-up, we calculated the number of patients who did not undergo DLS in our academic center after the introduction of DLS in the preoperative work-up for CRS + HIPEC (e.g. cohort B). Reasons for refraining from DLS were retrospectively explored from digital medical records.

Major postoperative complications are defined as grade 3 or higher according to the Clavien–Dindo classification system, and registered up to 90 days after surgery.^24^ These types of complications require endoscopic, radiologic, or surgical interventions, or admission to the intensive care unit. Postoperative mortality is defined as death within 30 days after surgery; OS is defined as the time between the initial exploratory laparotomy and death or date of last follow-up in censored cases; and DFS was defined as the time between CRS + HIPEC and the date of first recurrence or last follow-up in censored cases.

### Preoperative Evaluation and Staging

All referred patients with colorectal PM underwent a standardized preoperative evaluation to investigate the extent and resectability of the peritoneal disease and to rule out other distant metastases. All patients were staged with a computed tomography (CT) of the thorax, abdomen, and pelvis. Since 2012, laparoscopic evaluation in our academic center has been part of the preoperative work-up for CRS + HIPEC to further assess the extent of colorectal PM and the possibility of performing a complete cytoreduction. Patients with an absolute contraindication for CRS + HIPEC on imaging (i.e. extra-abdominal metastases or more than three liver metastases) were directly referred to a medical oncologist and did not undergo DLS. These patients are not represented in this article as they were not scheduled for CRS + HIPEC.

Every laparoscopic evaluation was performed under general anesthesia and a pneumoperitoneum was established by using an optical trocar. In all cases, a 30° laparoscope was used and introduced through an umbilical port. One or two additional trocars were placed under direct vision according to the surgeon’s discretion. All 13 abdominopelvic regions of the peritoneal cavity were systematically reviewed and adhesiolysis was only performed when deemed necessary. The laparoscopic PCI was calculated and the possibility to perform a complete cytoreduction during an exploratory laparotomy was estimated. The visibility of each abdominopelvic region, the laparoscopic PCI, and the possibility to achieve a complete cytoreduction were all recorded in the operation report. Cytology samples and biopsies were only taken as indicated. During several expert sessions with our four HIPEC surgeons, we created a 4-point scale for the degree of visibility of the abdominal cavity during DLS (i.e. grade I: visibility of two or less abdominopelvic regions; grade II: visibility of three to eight abdominopelvic regions; grade III: visibility of at least the diaphragm regions, pelvis region, and small bowel regions; and grade IV: visibility of all 13 abdominopelvic regions).

Hereafter, during a weekly multidisciplinary meeting, eligibility for CRS + HIPEC was determined by an experienced team consisting of medical oncologists, gastroenterologists, radiologists, and oncologic surgeons. In general, patients with colorectal PM were considered eligible for CRS + HIPEC when they met the following criteria: (1) PCI ≤ 20; (2) resectable primary tumor; (3) absence of extra-abdominal metastases; (4) absence of massive peritoneal disease involvement of the small bowel or its mesentery; (5) Eastern Cooperative Oncology Group (ECOG) performance status ≤ 3; and (6) American Society of Anesthesiologists (ASA) score of < 3. Up to three resectable liver metastases were not considered a contraindication for CRS + HIPEC.

### Cytoreductive Surgery with Hyperthermic Intraperitoneal Chemotherapy

CRS + HIPEC was performed according to the Dutch protocol.^3^ In summary, CRS was performed only in patients with completely resectable colorectal PM, and HIPEC was performed only after reaching a complete or nearly complete cytoreduction.

Each procedure started with an exploratory laparotomy to calculate the PCI score and judge the resectability of the colorectal PM. The procedure was terminated in cases where the patient was deemed not suitable for CRS + HIPEC, and palliative surgery was performed only according to the surgeon’s discretion (e.g. non-therapeutic laparotomy). Patients with resectable colorectal PM underwent CRS with the aim of removing all visible tumor tissue. The resection status after CRS was judged with the completeness of cytoreduction (CC) score.^25^ CC-0 indicates no visible or palpable residual tumor tissue in the peritoneal cavity; CC-1 indicates residual tumor deposits < 2.5 mm; CC-2 indicates residual tumor deposits between 2.5 mm and 2.5 cm; and CC-3 indicates residual tumor deposits > 2.5 cm, or confluence of unresectable tumor deposits at any site within the abdomen or pelvis.

HIPEC was performed in the case of a complete (CC-0) or nearly complete (CC-1) cytoreduction, whereby the abdominal cavity was perfused with mitomycin C (35 mg/m^2^) according to the open ‘Coliseum’ technique, with a temperature of 40–41 °C for 90 min.^26^ After HIPEC, reconstruction surgery, including bowel anastomoses, and, if deemed necessary, a colostomy, was performed. All patients were admitted to the intensive care unit for at least 1 postoperative day until cardiac and pulmonary functions were normal.

### Follow-Up

Clinical follow-up occurred within 1 month after surgery and thereafter on a quarterly basis for a minimum of 5 years. In the case of suspected recurrence based on clinical symptoms or an increase in carcinoembryonic antigen (CEA) level, a CT of the thorax and abdomen was performed.

### Data Collection

Relevant data were prospectively collected in an institutional database and consisted of patient characteristics, tumor characteristics, extent of peritoneal disease, previous treatments, operative characteristics, postoperative mortality and morbidity, and short- and long-term survival outcomes.

Reasons to refrain from DLS after its introduction in 2012 were retrospectively explored from digital medical records.

### Statistical Analyses

All statistical analyses were conducted using SPSS Statistics version 24.0 (IBM Corporation, Armonk, NY, USA). Categorical variables are reported as number (*n*) and percentages (%) and were analyzed using the Chi square test or Fisher’s exact test. Continuous variables are reported as median [interquartile range (IQR)] or mean ± standard deviation (SD) and were analyzed using the Student’s *t* test or Mann–Whitney *U* test. Kaplan–Meier survival analyses were performed to describe DFS and OS for study cohorts A and B. All tests were performed two-sided, and a *p* value < 0.05 was considered statistically significant.

## Results

### Baseline Characteristics

One hundred and seventy-two patients with histologically proven colorectal PM underwent an exploratory laparotomy for potential CRS + HIPEC in our academic center between January 2006 and January 2019. Forty-eight patients (27.9%) underwent an exploratory laparotomy before the introduction of DLS in the preoperative work-up for CRS + HIPEC (i.e. cohort A), and 124 patients (72.1%) underwent an exploratory laparotomy after the introduction of DLS in the preoperative work-up for CRS + HIPEC (i.e. cohort B). Table 1 shows a comparison of patient and tumor characteristics between cohorts A and B. Patients from cohort B, on average, were older (62 vs. 55 years; *p* < 0.002) and had a higher body mass index (BMI; 26.6 vs. 23.4 kg/m^2^; *p* < 0.001). Furthermore, these patients were less frequently diagnosed with an N2 (41.1 vs. 45.8%; *p* = 0.024) or M1 status (50.0 vs. 77.1%; *p* = 0.004) and were less frequently treated with adjuvant chemotherapy (25 vs. 41.7%; *p* = 0.001). On the other hand, patients from cohort B were more frequently diagnosed with metachronous onset of colorectal PM (54.0 vs. 33.3%; *p* = 0.015). Other baseline characteristics were similar between the cohorts.

**Table 1:** Baseline characteristics from all patients with colorectal PM who underwent an exploratory laparotomy for potential CRS + HIPEC, stratified by the operation date (cohort A: between 2006 and 2011; cohort B: between 2012 and 2019)

	Cohort A (*n* = 48)	Cohort B (*n* = 124)	*p* value
*Patient characteristics*			
Age, years (mean ± SD)	55.0 ± 9.7	62 ± 9.9	**0.002**
Female sex	22 (45.8)	60 (48.4)	0.764
BMI, kg/m^2^ (mean ± SD)	23.4 ± 4.7	26.6 ± 4.7	< **0.001**
ASA			0.871
1	6 (12.5)	19 (15.3)	
2	37 (77.1)	91 (73.4)	
3	5 (10.4)	14 (11.3)	
Comorbidity			
Diabetes mellitus	4 (8.3)	11 (8.9)	0.379
Hypertension	7 (14.6)	26 (21.0)	0.256
Cardiac comorbidity	7 (14.6)	12 (9.7)	0.878
Lung comorbidity	7 (14.6)	13 (10.5)	0.206
*Tumor characteristics*			
Primary tumor location			0.455
Right colon	23 (47.9)	41 (33.1)	
Transverse colon	2 (4.2)	10 (8.1)	
Left colon	4 (8.3)	15 (12.1)	
Sigmoid	13 (27.1)	40 (32.3)	
Rectum	6 (12.5)	18 (14.5)	
Signet cell histology	4 (8.3)	12 (9.7)	0.759
T-stage primary tumor			0.087
≤ 3	18 (37.5)	56 (45.2)	
4	25 (52.1)	66 (53.2)	
N status primary tumor			**0.024**
0	7 (14.6)	35 (28.2)	
1	14 (29.2)	36 (29.0)	
2	22 (45.8)	51 (41.1)	
M status primary tumor			
0	9 (18.8)	57 (46.0)	**0.004**
1	37 (77.1)	62 (50.0)	
Onset of colorectal PM			
Synchronous	32 (66.7)	57 (46.0)	**0.015**
Metachronous	16 (33.3)	67 (54.0)	
Synchronous liver metastases	4 (8.3)	12 (9.7)	0.785
*Prior CRC treatments*			
Prior CRC surgery	42 (87.5)	112 (90.3)	0.588
Prior chemotherapy	14 (29.2)	48 (38.7)	0.360
Neoadjuvant chemotherapy	4 (8.4)	24 (19.4)	0.568
Adjuvant chemotherapy	20 (41.7)	31 (25.0)	**0.001**

Bold values are statistically significant (*p* < 0.05)

Data are expressed as *n* (%) unless otherwise specified

*PM* peritoneal metastases, *CRS* cytoreductive surgery, *HIPEC* hyperthermic intraperitoneal chemotherapy, *SD* standard deviation, *BMI* body mass index, *ASA* American Society of Anesthesiologists, *synchronous onset* PM diagnosed at the time of presentation with colorectal cancer, *metachronous onset* PM diagnosed after initial curative colorectal resection, *CRC* colorectal cancer, *prior chemotherapy* chemotherapy used in the past, *neoadjuvant chemotherapy* chemotherapy prior to CRS with HIPEC, *adjuvant chemotherapy* chemotherapy after CRS with HIPEC

### Non-therapeutic Laparotomies

Table 2 presents the surgical characteristics of the exploratory laparotomy and postoperative morbidity rates for cohorts A and B.

**Table 2:** Treatment characteristics from all patients with colorectal PM who underwent an exploratory laparotomy for potential CRS + HIPEC, stratified by the operation date (cohort A: between 2006 and 2011; cohort B: between 2012 and 2019)

	Cohort A (*n* = 48)	Cohort B (*n* = 124)	*p* value
DLS routinely performed, yes	0 (0.0)	85 (68.5)	< **0.001**
HIPEC type			**0.044**
Open CRS + HIPEC	31 (64.6)	98 (79.0)	
Open–close procedure	17 (35.4)	26 (21.0)	
Main reason for the open–close procedure			0.496
PCI > 20	8 (47.1)^a^	13 (50.0)	
Too much small bowel involvement^b^	4 (23.5)	4 (15.4)	
Irresectable primary tumor^c^	2 (11.8)	7 (26.9)	
Irresectable liver metastases	3 (17.6)	2 (7.7)	
PCI at HIPEC			0.121
0–5	4 (36.4)	34 (28.8)	
6–10	2 (18.2)	26 (22.0)	
11–15	0 (0.0)	20 (16.9)	
16–20	0 (0.0)	16 (15.0)	
21–25	3 (27.3)	13 (11.0)	
> 25	2 (18.2)	9 (7.6)	
Total anatomic resections [median (IQR)]	4 (1–6)	4 (2–7)	0.410
Anastomoses			0.161
0	31 (64.6)	57 (46.0)	
1	12 (25.0)	44 (35.5)	
≥ 2	5 (10.5)	23 (18.5)	
Stoma post HIPEC	21 (43.8)	63 (50.8)	0.406
Operation time, min [median (IQR)]	493 (364–614)	471 (352–538)	0.217
Blood loss, mL [median (IQR)]	700 (475–1325)	750 (500–1500)	0.790
Resection status			0.126
CC-0 or CC-1	31 (64.6)	98 (79.0)	
≥ CC-2	17 (35.4)	26 (21.0)	
Length of hospital stay, days [median (IQR)]	15 (10–21)	16 (12–24)	0.239
Reoperation	4 (8.3)	15 (12.1)	0.480
In hospital mortality	1 (2.1)	2 (1.6)	0.833
Complication rate, Clavien–Dindo grade			0.424
I	4 (8.3)	10 (8.1)	
II	14 (29.2)	40 (32.3)	
III	7 (14.6)	23 (18.5)	
IV	7 (14.6)	6 (4.8)	

Bold values are statistically significant (*p* < 0.05)

Data are expressed as *n* (%) unless otherwise specified

*PM* peritoneal metastases, *CRS* cytoreductive surgery, *HIPEC* hyperthermic intraperitoneal chemotherapy, *DLS* diagnostic laparoscopy, *PCI* peritoneal cancer index, *IQR* interquartile range*, CC* completeness of cytoreduction

^a^During study period A (2006–2011), the PCI classification system was not used systematically in The Netherlands. In five patients from Cohort A with an open–close procedure, we concluded that the PCI would most likely have been above 20 based on the information from the operation report (i.e. extensive disease involvement of all nine abdominal regions)

^b^Massive peritoneal disease involvement of the small bowel or its mesentery, whereby removal will very likely lead to short bowel syndrome

^c^Tumor intertwined with vital structures, making safe removal impossible

None of the patients from cohort A underwent DLS during the preoperative work-up for CRS + HIPEC as it was not common clinical practice between 2006 and 2011. An unexpectedly low number of patients (85, 68.5%) underwent DLS in our academic center after the introduction of DLS in the preoperative work-up for CRS + HIPEC The number of non-therapeutic laparotomies for the entire cohort was 43 (25.0%). A non-therapeutic laparotomy occurred less frequently in cohort B when compared with historical cohort A (21.0 vs. 35.4%; *p* = 0.044). Causes for the occurrence of a non-therapeutic laparotomy did not differ between both cohorts (*p* = 0.496).

As the number of patients who underwent DLS in cohort B was unexpectedly low, additional analyses were performed to identify the direct effect of DLS on the prevention of non-therapeutic laparotomies. In this specific case, patients were no longer divided by their operation date (e.g. cohort A or B), but by whether they underwent DLS (*n* = 89) or not (*n* = 83). Non-therapeutic laparotomies occurred less frequently in patients who underwent DLS compared with patients who did not undergo DLS (18.0 vs. 32.5%; *p* = 0.028).

### Reasons to Refrain from Diagnostic Laparoscopy

An overview of the reasons to refrain from DLS for patients in cohort B after the introduction of the preoperative work-up for CRS + HIPEC is presented in Table 3. Refraining from DLS in our academic center was most frequently caused by the fact that the patient recently underwent a laparotomy (30.8%) or DLS (17.9%) in the referring hospital, or a laparotomy in our own academic center (17.9%). For these patients, in the decision-making process additional DLS in our academic center after recent abdominal surgery was not considered useful. Furthermore, DLS was not performed in seven patients (17.9%) who showed a clear response to neoadjuvant chemotherapy on CT imaging. In six patients (15.4%), reasons to refrain from DLS could not be identified from the digital medical records.

**Table 3:** Reasons for not routinely performing DLS in patients with colorectal PM from cohort B (*n *= 39)

Reasons for not routinely performing DLS	
Recent laparotomy in another hospital (< 4 weeks)	12 (30.8)
Recent DLS in another hospital (< 4 weeks)	7 (17.9)
Recent laparotomy in our academic center (< 4 weeks)	7 (17.9)
Clear response on neoadjuvant therapy on imaging	7 (17.9)
Unknown	6 (15.4)
*Impact on open*–*close procedures*	
HIPEC type	
Open CRS + HIPEC	28 (71.8)
Open–close procedure	11 (28.2)
Main reason for the open–close procedure	
PCI > 20	5 (45.5)
Too much small bowel involvement	2 (18.2)
Irresectable primary tumor	3 (27.3)
Irresectable liver metastases	1 (9.1)

Data are expressed as *n* (%)

*DLS* diagnostic laparoscopy, *PM* peritoneal metastases, *Cohort B* patients who underwent an exploratory laparotomy for potential CRS + HIPEC between January 2012 and January 2019, *CRS* cytoreductive surgery, *HIPEC* hyperthermic intraperitoneal chemotherapy, *PCI* peritoneal cancer index

Interestingly, in patients who did not undergo DLS after its introduction in the preoperative work-up for CRS + HIPEC, a non-therapeutic laparotomy occurred in 11 patients (28.2%). The specific reason for refraining from DLS was not predictive of an occurrence of a non-therapeutic laparotomy (*p* = 0.437) [data not shown]. There seemed to be a trend toward an increase in non-therapeutic laparotomies in patients from cohort B who did not undergo DLS compared with patients from the same cohort who underwent DLS in the preoperative work-up (28.2 vs. 17.6%), but this trend did not reach significance (*p* = 0.107).

### Laparoscopic Evaluation

Table 4 presents the surgical characteristics of the DLS and postoperative morbidity rates of the 85 patients (68.5%) from cohort B who underwent DLS prior to exploratory laparotomy. Good laparoscopic evaluation of the abdominal cavity (i.e. grade 3 or 4) was possible in 64 patients (74.1%). The conversion rate during DLS amounted to 21.2%, and no reoperations occurred. The postoperative complication rate was low (3.5%) and consisted only of Clavien–Dindo grade II complications (e.g. urinary tract infection and bacteremia). In patients who underwent DLS in the preoperative work-up for CRS + HIPEC, only 15 non-therapeutic laparotomies (17.6%) occurred.

**Table 4:** Visibility and postoperative morbidity of DLS in patients with colorectal PM from cohort B (*n* = 85)

*Time intervals*	
Interval of colorectal PM to DLS, months [median (IQR)]	1 (0–2)
Interval of colorectal PM to HIPEC, months [median (IQR)]	2 (2–4)
*Visibility during DLS*	
Grade of visibility^a^	
I (very poor)	11 (12.9)
II (poor)	8 (9.4)
III (good)	11 (12.9)
IV (excellent)	53 (62.4)
Conversion rate	18 (21.2)
PCI at DLS	
0–5	22 (25.9)
6–10	19 (22.4)
11–15	17 (20.0)
16–20	10 (11.8)
21–25	5 (5.9)
> 25	4 (4.7)
*Recovery after DLS*	
Length of hospital stay, days [median (IQR)]	2 (2–3)
Reoperation	0 (0.0)
Complication rate, Clavien–Dindo grade	
I	0 (0.0)
II	3 (3.5)
III	0 (0.0)
IV	0 (0.0)
Complication type	
Urinary tract infection	2 (2.4)
Bacteremia with unknown cause	1 (1.2)
*Impact on open*–*close procedures*	
HIPEC type	
Open CRS + HIPEC	70 (82.4)
Open–close procedure	15 (17.6)

Data are expressed as *n* (%) unless otherwise specified

*DLS* diagnostic laparoscopy, *PM* peritoneal metastases, *Cohort B* patients who underwent an exploratory laparotomy for potential CRS + HIPEC between January 2012 and January 2019, *IQR* interquartile range, *PCI* peritoneal cancer index, *CRS* cytoreductive surgery, *HIPEC* hyperthermic intraperitoneal chemotherapy

^a^Grade I: visibility of two or less abdominopelvic regions; grade II: visibility of three to eight abdominopelvic regions; grade III: visibility of at least the diaphragm regions, pelvis region, and small bowel regions; grade IV visibility of all 13 abdominopelvic regions

### Surgical Morbidity and Mortality

Table 2 presents the surgical characteristics of the exploratory laparotomy, along with the postoperative morbidity rates for cohorts A and B. One hundred and twenty-nine patients (75.0%) underwent CRS + HIPEC during an exploratory laparotomy. Treatment characteristics, consisting of the number of anatomic resections, PCI score, operating time, blood loss, and resection status, were similar for both cohorts.

Major postoperative complications after exploratory laparotomy occurred in 14 patients (29.2%) from cohort A and 29 patients (23.4%) from cohort B (*p* = 0.424). Relaparotomy was necessary in 4 (8.3%) and 15 patients (12.1%), respectively (*p* = 0.480). Overall in-hospital mortality was 1.7% and did not differ between the cohorts (*p* = 0.833).

### Survival Outcomes

The mean OS for the entire group of patients was 30.1 months [95% confidence interval (CI) 26.0–34.2 months], and the mean OS was similar for cohorts A and B [25.9 months (95% CI 19.5–32.3) vs. 29.5 months (95% CI 25.9–33.1 months); *p* = 0.132].

For additional analyses of OS and DFS after CRS + HIPEC, patients with a non-therapeutic laparotomy were excluded (*n* = 43). The mean OS for patients after CRS + HIPEC was 36.4 months (95% CI 31.6–41.2 months), and the mean OS was similar for cohorts A and B [34 months (95% CI 25.9–42.1 months) vs. 34 months (95% CI 30.2–37.8 months); *p* = 0.523]. The mean DFS for the entire cohort of patients was 20.7 months (95% CI 16.1–25.2 months), and the mean DFS was similar between cohorts A and B [20.9 months (95% CI 13.2–28.7 months) vs. 18.5 months (95% CI 14.7–22.4 months); *p* = 0.706].

## Discussion

In this observational study, consisting of 172 consecutive patients with colorectal PM, we demonstrated that non-therapeutic laparotomies during CRS occurred less frequently after the introduction of DLS as part of the preoperative work-up for CRS + HIPEC.

Proper selection of patients with colorectal PM for CRS + HIPEC is a known challenge as possible survival gain is difficult to weigh against treatment-related morbidity and mortality. From this perspective, for patients and clinicians, the most disappointing outcome after this major procedure is a non-therapeutic laparotomy as it is associated with an increased risk of postoperative morbidity and a diminished QoL without providing any improvement in survival. These days, up to 40% of patients with PM are still confronted with a non-therapeutic laparotomy during CRS.^15^^,^^16^ Previous research showed that DLS is an accurate and safe staging tool in patients with PM and might prevent non-therapeutic laparotomies in patients with extensive disease.^16^^,^^18^^–^23 In this study, we showed that the rate of non-therapeutic laparotomies significantly dropped from 35.4 to 21.0% after the introduction of DLS in our preoperative work-up, despite the fact that only 68.5% of patients underwent DLS in our academic center after this introduction. In the group of patients who underwent DLS, a trend towards an ever-lower rate of non-therapeutic laparotomies was found (17.6%). Additional analyses showed that recent abdominal surgery in two of three patients was the main reason to refrain from DLS in our academic center, resulting in an unexpectedly higher rate of non-therapeutic laparotomies (28.2%) in these patients. An explanation for this phenomenon might be the fact that surgeons from the referral centers in most cases were unexpectedly confronted with colorectal PM during a primary tumor resection. At that moment, the focus would be on referring the patient to a highly experienced HIPEC center as quickly as possible, and therefore less attention might be paid to the true extent of the peritoneal disease.

In our current study, some significant differences in baseline characteristics were found between patients who underwent an exploratory laparotomy for potential CRS + HIPEC before and after the introduction of DLS in the preoperative work-up for CRS + HIPEC (i.e. cohort A and B, respectively). Patients from cohort B were, on average, older and had a higher BMI, which can be explained by the increase in the global average life expectancy and the increase in obesity rates during the past 20 years. Both age and BMI are not considered a contraindication for CRS + HIPEC in our academic center. Patients from cohort B were also less frequently treated with adjuvant chemotherapy. Due to a lack of scientific evidence, there is no worldwide consensus about the use and timing of perioperative chemotherapy. Over the years, we have become more careful in applying adjuvant chemotherapy to patients after CRS + HIPEC because of the increase in morbidity and temporal decrease in QoL, which are both associated with chemotherapy. It is very unlikely that these differences in age, BMI, and the use of adjuvant chemotherapy could explain the rate drop of non-therapeutic laparotomies in cohort B. Furthermore, patients from cohort B were also more frequently diagnosed with metachronous onset of colorectal PM. The most likely explanation for this phenomenon seems the shift towards an increased awareness about CRS + HIPEC among surgeons from regional hospitals. In the past, patients with metachronous colorectal PM in particular were frequently referred to a medical oncologist for palliative treatment options instead of an experienced HIPEC center. These days, patients are referred to our academic center in a low-threshold way, resulting in the treatment of more patients with metachronous onset of colorectal PM. To the best of our knowledge, there are no scientific publications regarding the impact of the onset of colorectal PM on the rate of non-therapeutic laparotomies during CRS + HIPEC.

Overall, six other studies have reported data about the impact of DLS on preventing non-therapeutic laparotomies in patients with PM during CRS.^16^^,^^19^^–^23 It should be noted that none of these studies focused only on patients with colorectal PM; a variation of 3, up to 11, primary tumor types were included per study. The overall rate of non-therapeutic laparotomies during CRS in patients who underwent DLS ranged from 12.5 to 37.0%. In most studies, DLS was used in only highly selected patients.^19^^,^^21^^,^^22^ When DLS was routinely performed in all patients with PM, low rates of non-therapeutic laparotomies during CRS were reported (ranging from 15.2 to 17.0%).^20^^,^^23^ In only three studies was it possible to compare rates of non-therapeutic laparotomies between patients who underwent DLS and patients who did not undergo DLS prior to CRS.^16^^,^^19^^,^^22^ These studies all reported a significant drop in the rate of non-therapeutic laparotomies in patients who underwent DLS when compared with patients who did not undergo DLS prior to CRS. However, it remains challenging to compare the results from our present study with the current literature because of differences in patient populations, tumor types, and indications to perform DLS.

In The Netherlands, HIPEC procedures are only performed in highly experienced tertiary referral centers by a dedicated team of surgeons. As previously mentioned, most surgeons from referral centers have less experience in reporting the extent of colorectal PM according to the PCI score, and therefore might understage the extent of disease and overestimate the possibility of achieving a complete cytoreduction. With this obtained knowledge, we are paying more attention to early detection and referring of patients with colorectal PM to our academic center. Patients will undergo laparoscopic evaluation by one of our HIPEC surgeons to investigate the extent and resectability of the colorectal PM, independently of prior abdominal surgery performed at the referral center. With these adjustments, we suspect that the rate of non-therapeutic laparotomies in patients with colorectal PM will drop even further in our academic center in the following years.

In the near future, it is possible that DLS will play a smaller role in patient selection because detection rates of PM from current preoperative imaging modalities are improving.^27^^,^^28^ In a recent study consisting of 49 patients with colorectal PM, MRI PCI was strongly correlated with the surgical PCI.^28^ Two radiologists with extensive experience in detecting colorectal PM could identify all patients with resectable disease based on a PCI score below 21. Larger series are still necessary to provide more evidence of the accuracy of detection and staging of colorectal PM. DLS in the preoperative work-up for CRS + HIPEC will not be easily curbed as other advantages remain, such as taking biopsies to confirm the presence or absence of peritoneal disease and provide additional information for future systemic therapies.

This study has certain strengths and limitations. To the best of our knowledge, this is the first study that specifically describes the impact of DLS to prevent non-therapeutic laparotomies in a large cohort of patients with colorectal PM. Another strength of the current study is the presence of an adequate comparison group; an historical cohort of all consecutive patients who underwent an exploratory laparotomy for potential CRS + HIPEC before DLS was introduced in our academic center. Gathered knowledge from this study provided crucial information about our daily practice to further improve the implementation of DLS in the preoperative work-up for CRS + HIPEC. On the other hand, our study has some limitations due to its retrospective design and single-center approach. Selection bias might have occurred, although most data were obtained from a prospectively maintained institutional database, and reasons to refrain from DLS in a subset of patients were further investigated. Although our HIPEC surgeons are extensively trained to perform CRS + HIPEC procedures and already had extensive experience in gastrointestinal surgery, study results may have also been influenced by their learning curves in the beginning of this study period. Learning curves from our academic center and other Dutch hospitals have already been published elsewhere.^29^

## Conclusions

Non-therapeutic laparotomies during CRS (e.g. open–close procedures) are prevented in patients with colorectal PM when DLS is performed during the preoperative work-up for this major abdominal procedure. We recommend that only HIPEC surgeons perform this laparoscopic evaluation to ensure adequate staging of the extent of colorectal PM and the possibility of achieving a complete cytoreduction.
